# The Ecto-5’-Nucleotidase/CD73 Inhibitor, α,β-Methylene Adenosine 5’-Diphosphate, Exacerbates Carrageenan-Induced Pleurisy in Rat

**DOI:** 10.3389/fphar.2019.00775

**Published:** 2019-07-11

**Authors:** Elisabetta Caiazzo, Silvana Morello, Rosa Carnuccio, Armando Ialenti, Carla Cicala

**Affiliations:** ^1^Department of Pharmacy, School of Medicine, University of Naples Federico II, Via Domenico Montesano 49, Naples, Italy; ^2^Department of Pharmacy, University of Salerno, Via Giovanni Paolo II 132, Fisciano, SA, Italy

**Keywords:** adenosine, CD73, ecto-5’-nucleotidase, inflammation, pleurisy, rat

## Abstract

The ecto-5’-nucleotidase (ecto-5’NT/CD73) represents a crucial enzyme for endogenous adenosine generation. Several findings have shown that CD73 plays an important role in regulating vascular permeability and immune cell function. Adenosine 5’-(α,β-methylene)diphosphate (APCP) is a CD73 inhibitor, widely used as pharmacological tool to investigate the role of CD73/adenosine pathway in several *in vitro* and *in vivo* models, although it has been also shown to inhibit other ectoenzymes involved in adenosinergic pathway. Here, we evaluated the effect of APCP in the development of inflammation in carrageenan-induced pleurisy model. We found that treatment with APCP (400 µg/rat) significantly increased cell accumulation, exudate formation, and pro-inflammatory cytokine content into the pleural cavity in the acute phase (4 h) of inflammation, with no differences in the sub-acute phase (72 h) except for the regulation of monocyte chemotactic protein-1 levels. In addition, cells collected by pleural lavage fluids of APCP-treated rats, 4 h following carrageenan injection, showed increased ability to migrate *in vitro*, both in presence and in absence of N-formyl-L-methionyl-L-leucyl-L-phenylalanine as chemotactic stimulus, compared to cells obtained by control rats. Our results demonstrate that APCP exacerbates the early phase of carrageenan-induced pleurisy by controlling pleural effusion and polymorphonuclear migration *in vivo* and *ex vivo*. This effect is likely dependent upon CD73 inhibition, although an inhibitory effect of other ectoenzymes cannot be ruled out.

## Introduction

Ecto-5’-nucleotidase (ecto-5’NT/CD73), the key enzyme in leading to adenosine accumulation, has been localized on barrier cell types, such as endothelial cells, and participates in the control of barrier permeability. Thompson and coworkers (2004) by performing experiments on genetically modified mice, lacking CD73, were the first to demonstrate the important role for this ecto-enzyme in the control of the vascular leakage following hypoxia, a common feature of inflamed tissues. This finding was in agreement with previous experimental work demonstrating that following hypoxia the increased CD73 activity and extracellular adenosine accumulation represent a protection preserving intestinal epithelium from vascular leakage. On the other hand, administration to mice of the CD73 inhibitor α,β-methylene adenosine 5’-diphosphate (APCP) significantly increased the permeability of intestinal epithelium (Synnestvedt et al., 2002; Ledoux et al., 2003). Although CD73 has been shown to play an important role in the development of pulmonary inflammation and protection against lung injury induced by artificial ventilation in mice (Eckle et al., 2007), less is known on changes of its expression and activity on tissues and cells following inflammation. Indeed, in an inflammatory environment, recruited and stromal cells expressing CD73 are critical producers of adenosine that, in turn, by engaging its receptors on adjacent cells, exerts an immunomodulatory effect (Antonioli et al., 2013).

Pleurisy is characterized by inflammation of the pleura, the tissue composed of mesothelial cells, lining the lung and regulating pleural cavity homeostasis. Carrageenan-induced rat pleurisy is a model characterized by an early (4 h) and late (72 h) phase of inflammation, and it is a useful model to study pleural effusion, resulting in extravasation and cell accumulation into the pleural space (Murai et al., 2003) that may represent a severe complication of pulmonary and extra pulmonary diseases.

Aim of the present study was to analyze the role of CD73 in pleural effusion, in rat. We found that treatment with the CD73 inhibitor, APCP, influences the accumulation of leukocytes and cytokine production within pleural cavity. These effects were associated with worsened lung injury and enhanced ability of pleural leukocytes to migrate. All together, our data suggest that CD73 represents an endogenous modulator of pleural effusion during the early phase of inflammation.

## Materials and Methods

### Animals

All experiments were performed on male Wistar rats (220–260 g; Charles River, Calco, Italy). The animals were maintained at a room temperature of 22 ± 2° on a 12 h/12 h light/dark cycle and were housed in a specific pathogen-free environment and fed standard rodent chow and water *ad libitum*. All procedures were performed according to the Italian (DL 26/2014) and European (n.63/2010/UE) regulations on the protection of animals used for experimental and other scientific purposes and were approved by Italian Ministry of Health (Number 1039/2016).

### Carrageenan-Induced Pleurisy

Rats were anesthetized with 4% enflurane mixed with 0.5 L/min O_2_ and 0.5 L/min N_2_O, and a skin incision at the level of the left sixth intercostal space was performed. The underlying muscle was dissected, and 0.2 ml of λ-carrageenan type IV (1% w/v; Sigma-Aldrich, Milan, Italy) was injected into the pleural cavity. The CD73 inhibitor, APCP (400 μg/rat; Tocris Bioscience, Bristol, UK), or an equal volume of the vehicle (distilled water), was injected into the pleural cavity immediately before carrageenan injection. The skin was then sutured, and animals were returned to their cages and allowed to have food and water *ad libitum*; 4 and 72 h following pleurisy induction, rats were sacrificed by CO_2_ inhalation. The chest was carefully opened, and the pleural cavity was washed with 2 ml of sterile saline containing 10 U/ml heparin (Sigma-Aldrich, Milan Italy). Any lavage fluid with blood contamination was rejected. The volume of pleural lavage fluid collected from each animal was measured; then, fluids were centrifuged at 180 × g for 10 min and the pellet suspended in phosphate-buffered saline (PBS). Cells were counted with TC20^™^ Automated Cell Counter (Bio-Rad, Italy). Samples of supernatants and cell pellets were then frozen at −80°C to be successively analyzed for cytokine content and AMPase activity, whereas differential cell count was performed in smears by May-Grunwald-Giemsa staining (Carlo Erba, Italy). Lung samples were harvested from each rat and immediately frozen at −80°C or fixed in formaldehyde solution (4% v/v, in distilled water) for 1 week at room temperature and successively utilized for further analyses.

### Morphological Analysis

Morphological analysis was performed on pulmonary tissue samples from vehicle- and APCP-treated rats. Lung biopsies harvested 4 or 72 h following carrageenan injection and fixed in formaldehyde, as described above, were dehydrated using graded ethanol and embedded in Paraplast (Sherwood Medical, Mahwah, NJ). Tissue sections (7-μm thickness) were then deparaffinized with xylene and stained with hematoxylin and eosin (Kaltek, Padova, Italy). A minimum of five sections *per* animal was analyzed under direct light microscopy (original magnification X 20) and photographed by a Leica DFC320 video camera (Leica, Milan, Italy) connected to a Leica DM RB microscope by using the Leica Application Suite software V 4.1.0.

### AMPase Activity

AMPase activity, evaluated as previously described (Caiazzo et al., 2016), was assessed in cells, lung samples, and cell-free pleural lavage fluids collected from rats 4 or 72 h following pleurisy induction, by colorimetric measurement of the inorganic phosphate (P*i*) released following incubation with the substrate, as described by Nedeljkovic et al. (2006). Cell pellets were suspended in 50 μl of ice-cold lysis buffer [4-[2-hydroxyethyl]-1-piperazine ethane sulfonic acid (HEPES), 200 mM; NaCl, 400 mM; dithiothreitol (DTT), 1 mM; IGEPAL, 1%; glycerol, 20% (Carlo Erba, Italy), plus the protease inhibitor cocktail (Roche, Italy)] and maintained in constant agitation for 45 min at 4°C. Lung tissue was transferred into a tube preloaded with one (6.35 mm) diameter zirconium oxide coated ceramic grinding sphere in ice-cold lysis buffer [Tris-HCl, 50 mM pH 7.5; NaCl, 150 mM; sodium orthovanadate, 1 mM; β glycerophosphate, 20 mM; ethylenediaminetetraacetic acid (EDTA), 2 mM; phenylmethylsulfonyl fluoride (PMSF), 1 mM; leupeptin, 5 μg/ml; aprotinin, 5 μg/ml; pepstatin, 5 μg/ml; ICN Pharmaceuticals, Italy] and put into a FastPrep^®^-24 homogenizer (MP Biomedicals, Santa Ana, California, USA) for lysis. Cell lysates and lung homogenates were then centrifuged for 15 min at 8,000 × g at 4°C, and the supernatant (protein extract) was collected and stored at −80°C until analysis. To initiate the enzymatic reaction, samples (50 µg of proteins) were incubated with 200 μl of medium containing MgCl_2_ (10 mM), NaCl (120 mM), KCl (5 mM), glucose (60 mM), Tris-HCl (50 mM), and pH 7.4. After 10 min, AMP (2 mM) was added as substrate and samples kept at 37°C for 40 min. The reaction was then stopped by the addition of trichloroacetic acid (final concentration 5% w/v). Following sample centrifugation at 500 × g for 10 min, at 37°C, the release of P*i* was measured using Malachite Green Phosphate Assay Kit (ScienCell, Research Laboratories, Carlsbad, USA) and KH_2_PO_4_ as standard (Chan et al., 1986). To have the net value of P*i* produced following enzymatic reaction, aspecific P*i* released in the absence of AMP in each sample was evaluated and the value obtained was subtracted from the value obtained following incubation with AMP. Protein concentration was measured by Bradford assay, and results were expressed as P*i* released (pmol/min/µg protein). AMPase activity was also assessed in cell-free pleural lavage fluids collected from rats 4 and 72 h following pleurisy induction by performing the same procedure described above.

### Cytokine Measurement

In the pleural lavage fluids collected 4 and 72 h following carrageenan injection, levels of tumor necrosis factor-α (TNF-α), interleukin-1β (IL-1β), interleukin-6 (IL-6), and monocyte chemotactic protein-1 (MCP-1) were evaluated by a colorimetric commercial enzyme-linked immunosorbent assay (ELISA) kit (R&D Systems, Minneapolis, MN) according to the manufacturer’s instructions and expressed as pg *per* ml.

### Chemotaxis Assay

Chemotaxis was evaluated on cells obtained from pleural lavage fluids collected 4 h after carrageenan injection in a 48-well modified Boyden chamber (AP48, Neuro Probe, USA). Briefly, 2.50 × 10^5^ cells in Roswell Park Memorial Institute (RPMI-1640) medium containing 0.1% bovine serum albumin (BSA) (50 µl) were placed on top of the polycarbonate filter (8-µm pore size, Neuro Probe, USA) whereas 25 μl of chemoattractant N-formyl-L-methionyl-L-leucyl-L-phenylalanine (fMLP, 1 ng/ml in RPMI-1640 medium containing 0.1% BSA) were added to the wells on the bottom. Spontaneous migration was determined using RPMI-1640 medium without fMLP. Each condition was set up in triplicate. Chambers were incubated for 90 min at 37°C in humidified air containing 5% CO_2_. Following incubation, the chamber was disassembled and the filter was carefully removed and washed with sterile PBS. Cells that failed to migrate through the filter were wiped off the top surface of the filter. Migrated cells on the filter were fixed and stained with 4’,6-diamidino-2-phenylindole (DAPI, Vector Laboratories, Burlingame, ON, Canada), and migration was quantified as the total pixel count of DAPI-stained nuclei under the fluorescence microscope using Fiji software. Migration index of cells obtained from pleural lavage fluids, harvested from vehicle- and APCP-treated rats, was expressed as ratio between the number of migrated cells in presence of fMLP and the number of migrated cells in the absence of fMLP.

### Flow Cytometry

Pleural lavage fluids recovered from the pleural cavity were washed twice in saline, and the resulting suspensions were pelleted by centrifugation at 180 × g for 10 min at 4°C. To block non-specific Fc-mediated interactions, cell samples were pre-incubated with an FcR blocking reagent (BD Biosciences) on ice for 10 min and then incubated with the following antibodies against: CD11b/c-PerCP/Cy5.5 (clone OX-42, BioLegend), RP-1-PE (BD Pharmingen), CD68-FITC (clone ED1, GeneTex), and CD73-Alexa Fluor 647 (Bioss Antibodies) for 20 min in the dark. Data were acquired with a FACSCalibur flow cytometer and analyzed with CellQuest software (BD FACSCalibur, Milan, Italy).

### Statistical Analysis

All data are presented as mean ± standard error (SE); statistical analysis was performed on raw data by two tailed Student’s t-test for unpaired data or by one-way ANOVA followed by Bonferroni *post-hoc* test as appropriate. A p value < 0.05 was considered statistically significant.

## Results

### APCP Treatment Increases Pleural Effusion and Cell Infiltration

**Figure 1:**
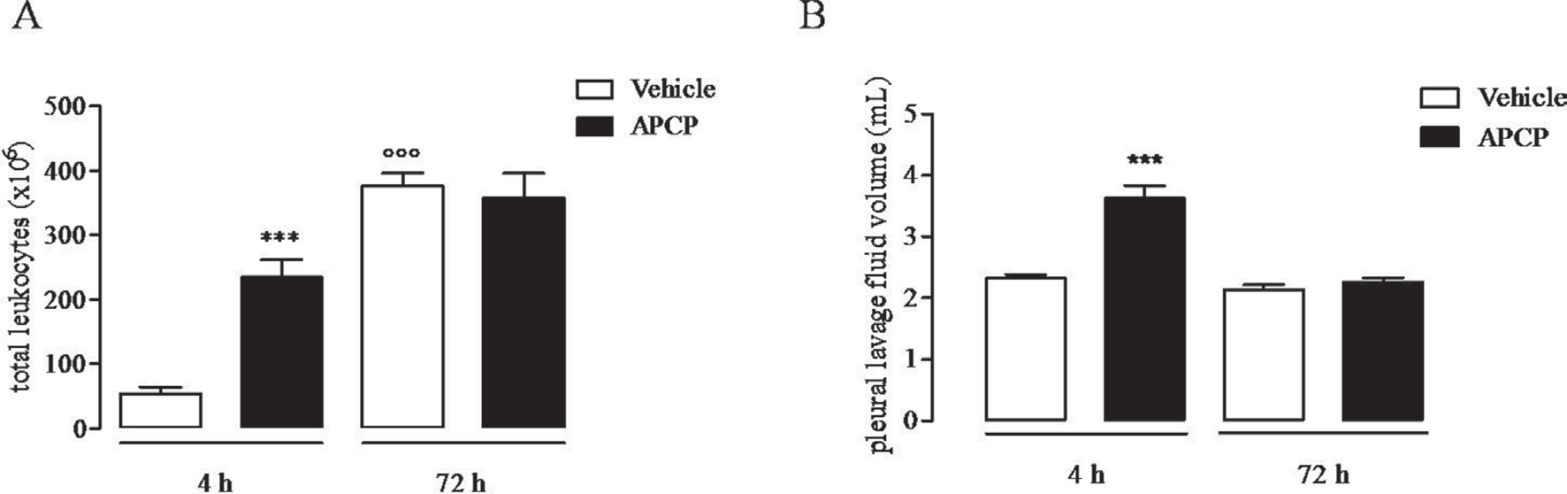
Effect of APCP on leukocyte infiltration and pleural lavage fluid volume 4 and 72 h following carrageenan-induced rat pleurisy. **(A)** Total leukocytes. **(B)** Pleural lavage fluid volume. Data are expressed as mean ± SE, N = 10; **(A)** ***p < 0.001 and °°°p < 0.001 *versus* vehicle 4 h. **(B)** ***p < 0.001 *versus* vehicle 4 h. Two-tailed Student’s t-test.

Treatment of rats with CD73 inhibitor, APCP (400 μg/rat), significantly increased cell accumulation into the pleural cavity 4 h following carrageenan injection compared to vehicle (**Figure 1A**), while the leukocyte number into the pleural lavage fluids collected at 72 h after carrageenan injection was similar in APCP- and vehicle-treated rats (**Figure 1A**). The volume of exudate collected by pleural lavage 4 h following carrageenan injection significantly increased in APCP-treated rats compared with control (vehicle-treated) rats (**Figure 1B**). There was no difference in the volume of exudate produced at 72 h after carrageenan injection between APCP- and vehicle-treated rats (**Figure 1B**).

### APCP Treatment Increases Lung Damage

**Figure 2:**
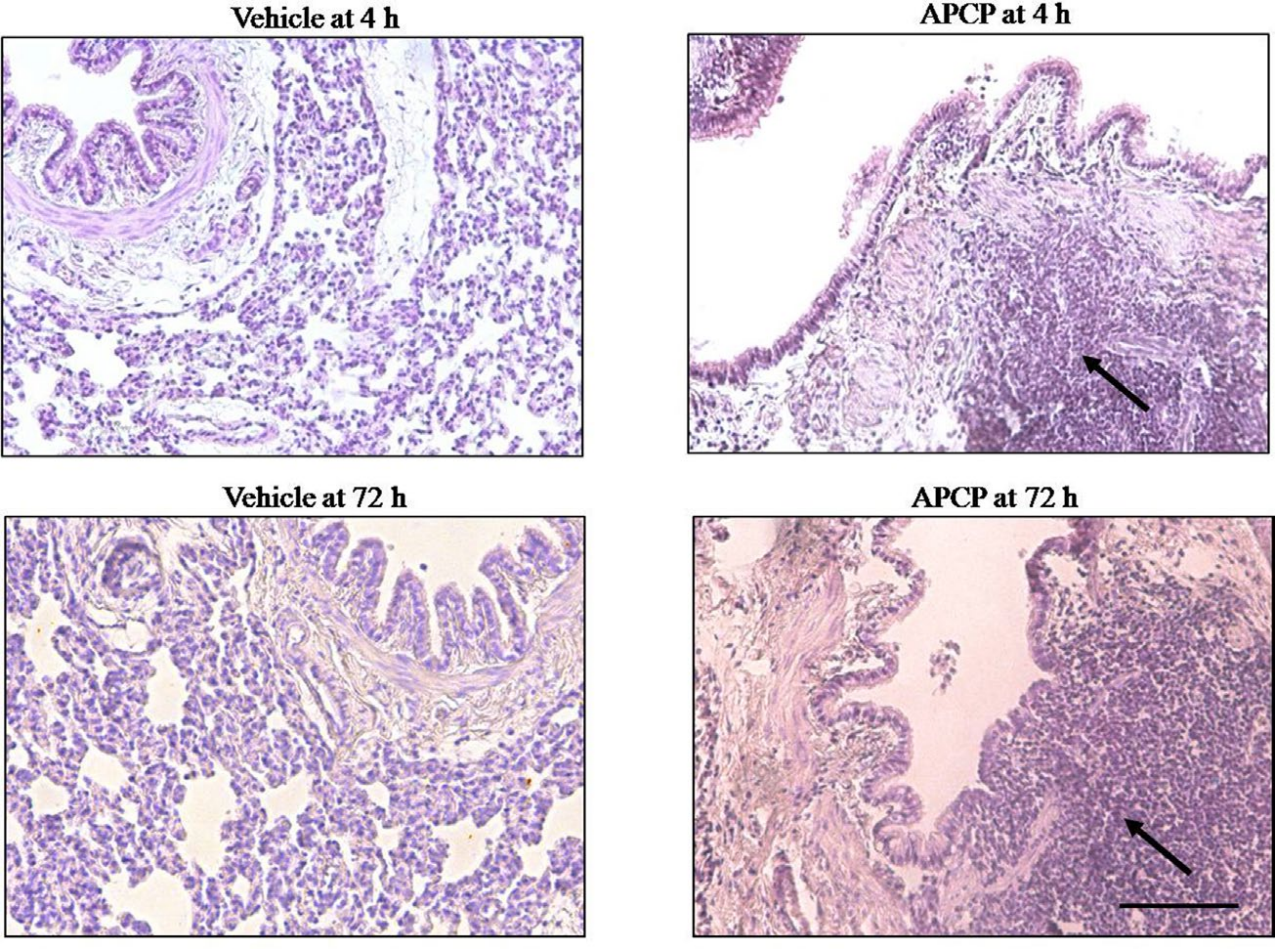
Effect of APCP on lung injury 4 and 72 h following carrageenan-induced pleurisy. Representative morphological analysis of hematoxylin and eosin (H&E)-stained lung sections from vehicle group (4 and 72 h) and APCP group (4 and 72 h). Black arrows indicate points of intense inflammatory cell infiltration. Original magnification X 20. Scale bar = 100 µm.

Morphological analysis of inflamed lung sections showed cell infiltration into bronchial and perivascular space as well as lung injury (**Figure 2**). The increased inflammatory cell infiltration in APCP-treated rats was associated with damaged lung architecture 4 and 72 h following carrageenan-induced pleurisy (**Figure 2**).

### APCP Treatment Is Associated With Reduced AMPase Activity

**Figure 3:**
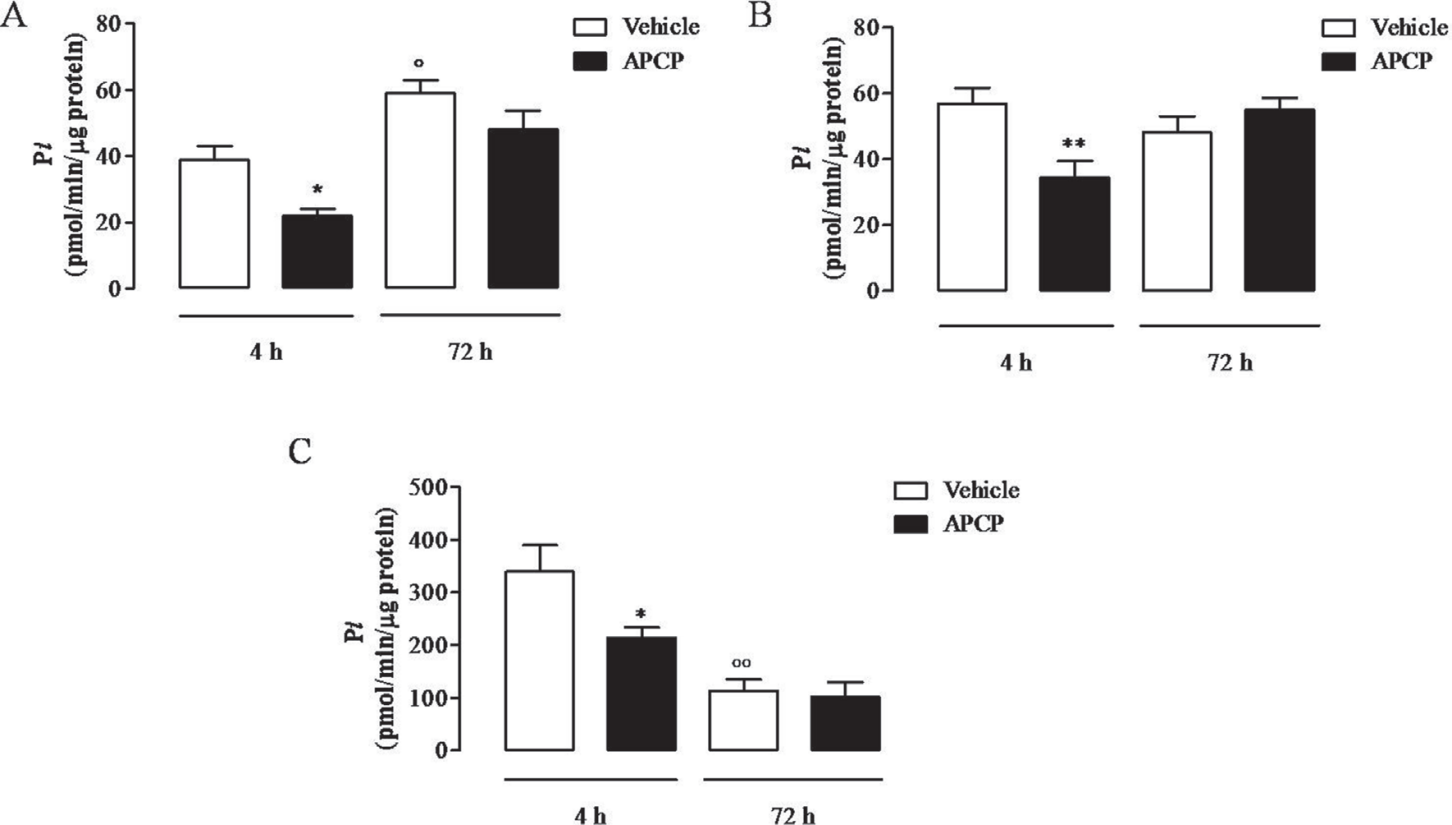
AMPase activity in cells **(A)**, lungs **(B)**, and pleural lavage fluids **(C)** collected 4 and 72 h following carrageenan-induced pleurisy. Data are expressed as mean ± SE, N = 6; **(A)** *p < 0.05 and °p < 0.05 *versus* vehicle 4 h. **(B)** **p < 0.01 *versus* vehicle 4 h. **(C)** *p < 0.05 and °°p < 0.01 *versus* vehicle 4 h. Bonferroni’s multiple comparison test.

In APCP-treated rats, the AMPase activity in cell lysates, in lung homogenates, and in cell-free pleural lavage fluids 4 h following carrageenan injection was significantly reduced compared with vehicle, but not at 72 h (**Figures 3A, B, and C**, respectively). It is worth noting that the AMPase activity in cell lysates at 72 h was higher than at 4 h (**Figure 3A**) and in cell-free pleural lavage fluids was lower at 72 h than at 4 h (**Figure 3C**). In lung homogenates, there was no difference in AMPase activity between 4 and 72 h (**Figure 3B**).

### APCP Treatment Increases Cytokine Production in Pleural Fluids

**Figure 4:**
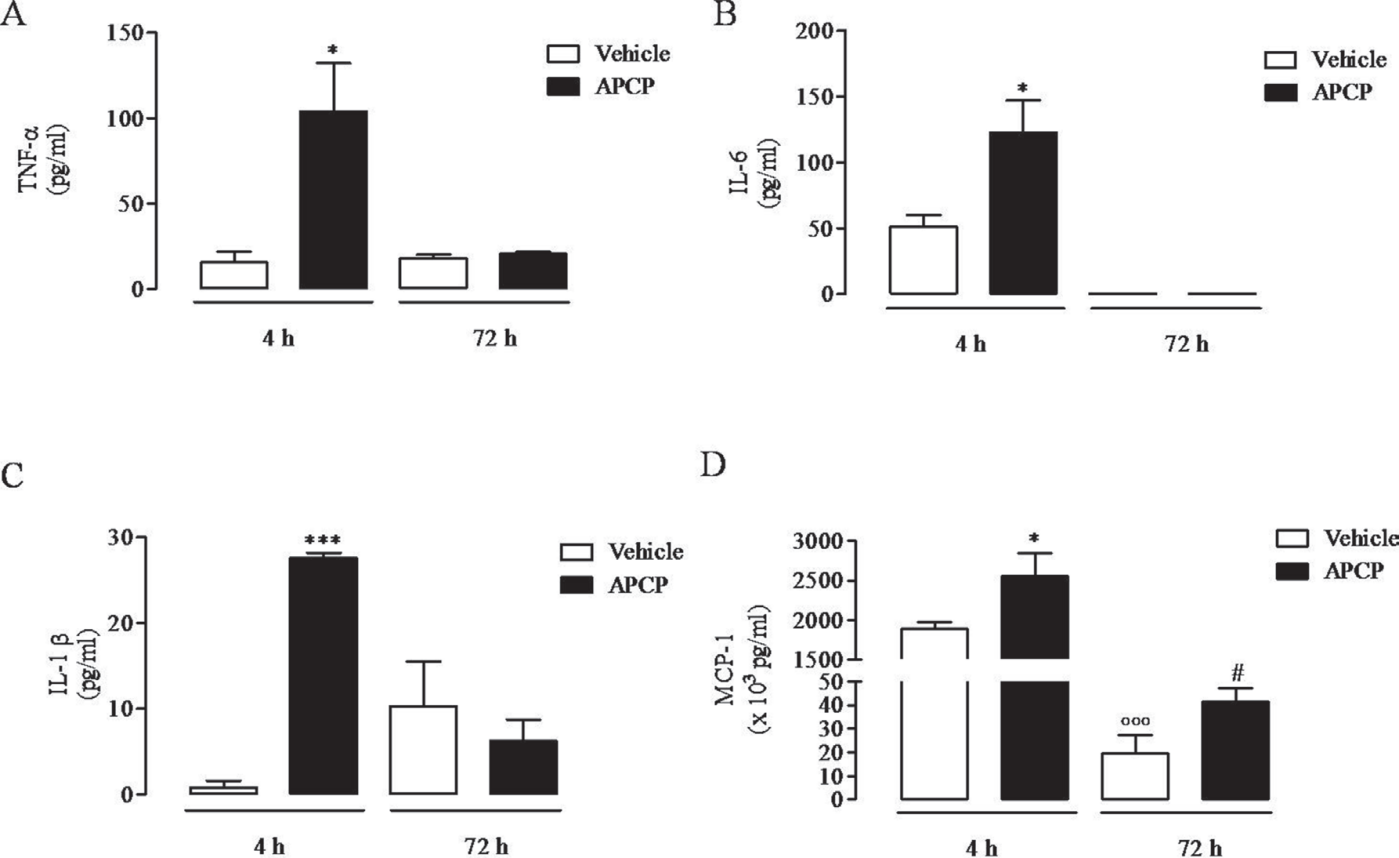
Effect of APCP on levels of TNF-α **(A)**, IL-6 **(B)**, IL-1β **(C)**, and MCP-1 **(D)** in pleural lavage fluid recovered from the pleural cavity 4 and 72 h following carrageenan-induced rat pleurisy. Data are expressed as mean ± SE, N = 6; **(A)** *p< 0.05 *versus* vehicle 4 h. **(B)** *p< 0.05 *versus* vehicle 4 h. **(C)** ***p < 0.001 *versus* vehicle 4 h. **(D)** *p < 0.05 and °°°p < 0.001 *versus* vehicle 4 h; ^#^p < 0.05 *versus* vehicle 72 h. Two-tailed Student’s t-test.

Following treatment with APCP, there was a significant increase in TNF-α, IL-6, IL-1β, and MCP-1 levels evaluated in cell-free pleural lavage fluids collected 4 h following pleurisy induction (**Figures 4A, B, C, and D**, respectively). In contrast, TNF-α, IL-6, and IL-1β levels in pleural lavage fluids collected at 72 h were similar in APCP- and vehicle-treated rats (**Figures 4A, B, and C**, respectively), while MCP-1 levels were still significantly increased in APCP-treated rats compared with control (vehicle-treated) at 72 h (**Figure 4D**).

### APCP Treatment Increases PMN Migration Index

**Figure 5:**
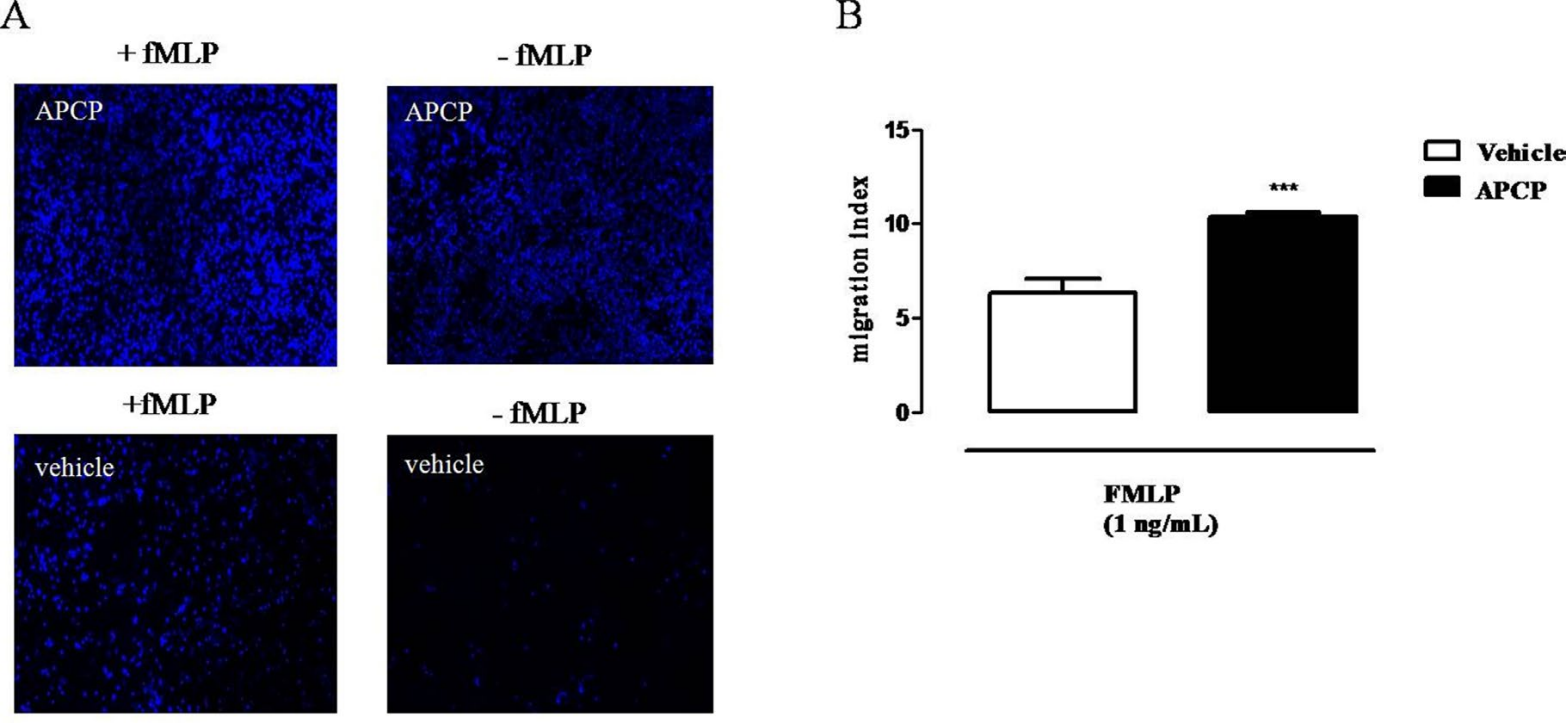
Effect of APCP on chemotaxis of leukocytes collected 4 h after carrageenan injection. **(A)** Representative immunofluorescence showing DAPI-stained nuclei from vehicle and APCP-treated group; cell chemotaxis was evaluated in presence and in absence of fMLP (see text for details). Original magnification X 10. **(B)** Histogram representing the results of migration index expressed as ratio between the number of migrated cells in presence of fMLP and the number of migrated cells without fMLP. Cells were collected from pleural lavage fluids harvested from vehicle- and APCP-treated rats. Data are expressed as mean ± SE, N = 8. ***p < 0.001 *versus* vehicle. Two-tailed Student’s t-test.

Cells obtained from pleural lavage fluids collected from APCP-treated group of rats 4 h after carrageenan injection showed increased ability to migrate *in vitro* both in presence and in absence of a chemotactic stimulus (fMLP), compared to cells from vehicle groups, as showed in the representative images of **Figure 5A**. Accordingly, the migration index was significantly increased in samples from APCP-treated rats compared with control (vehicle-treated rats) in presence of fMLP (**Figure 5B**).

### Leukocyte Populations into the Pleural Cavity

**Figure 6:**
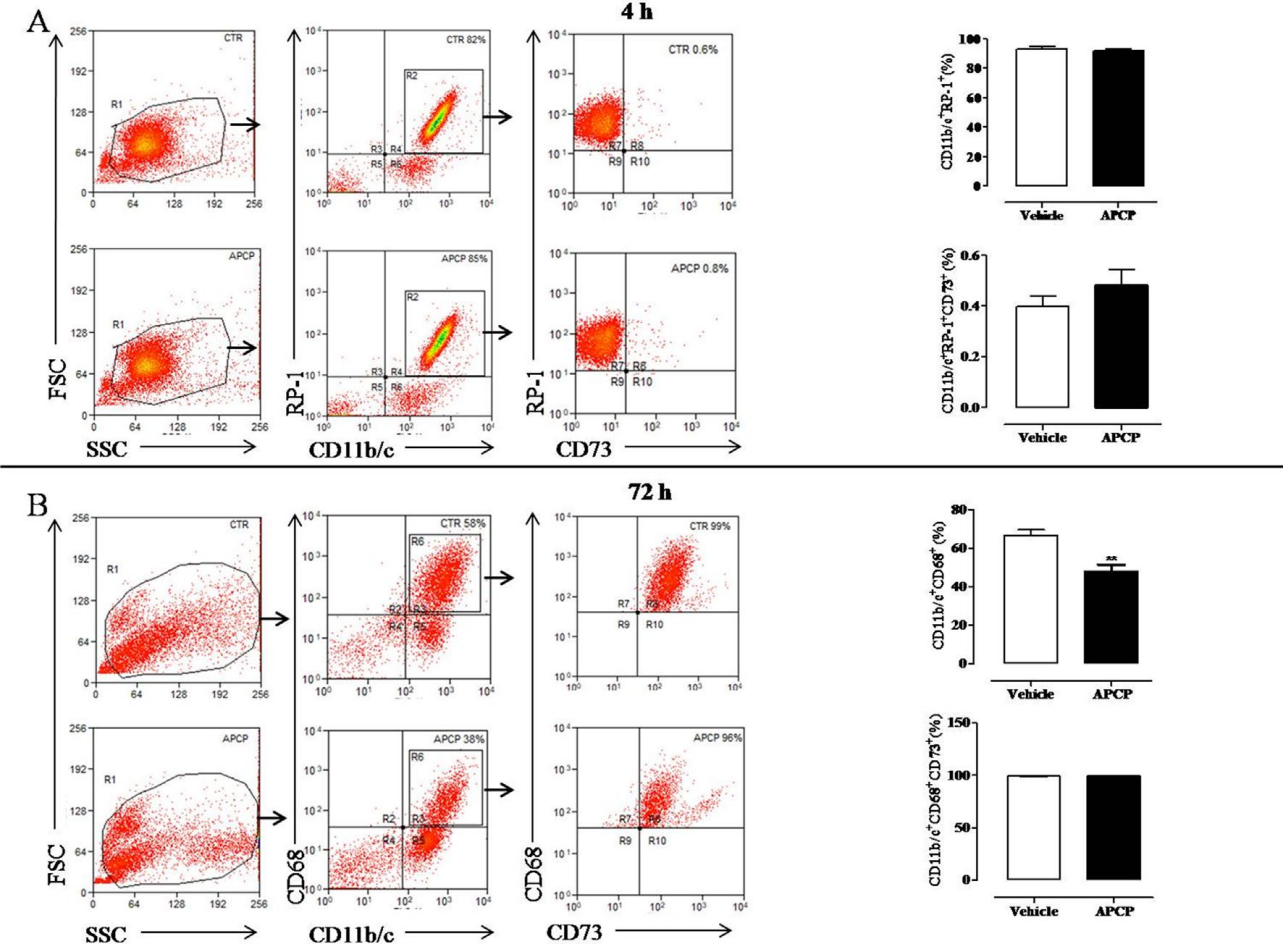
Analysis of CD73 expression on leukocyte subpopulations recovered from the pleural cavity at 4 h **(A)** and 72 h **(B)** after carrageenan injection. Flow cytometry dot plots in panel A (left) show a representative experiment illustrating the gating strategy used to analyze CD11b/c positive ^(+)^ RP-1 positive ^(+)^ cells gated on leukocytes population identified by forward and side scatter characteristics. The CD73 expression was analyzed on CD11b/c^+^ RP-1^+^ cells. On the right of panel A, histograms summarize the percentage of cells positive to CD11b/c and RP-1 and the percentage of CD73 positive cells among CD11b/c^+^ RP-1^+^ cells in vehicle and APCP groups. Data are expressed as mean ± SE, N = 7. In panel B, flow cytometry dot plots are representative of CD11b/c^+^ CD68^+^ cells within leukocyte population identified by forward and side scatter characteristics. CD11b/c^+^ CD68^+^ cells were then analyzed for their CD73 expression. On the right of panel B, histograms summarize the percentage of cells positive to CD11b/c and CD68 and the percentage of CD73 positive cells among CD11b/c^+^ CD68^+^ cells in vehicle and APCP groups. Results represent mean ± SE, N = 7. **p < 0.01. Two-tailed Student’s t-test.

Differential cell count of leukocytes migrated into the pleural cavity showed that PMN neutrophils dominated the early phase (4 h) of the reaction and were replaced by monocytes at 72 h (data not shown). To assay neutrophil and monocyte cell subsets and the expression of CD73, cells collected from pleural lavage fluids at 4 and 72 h following pleurisy induction were analyzed by ﬂow cytometry. Cells collected at 4 hours were mostly neutrophils (CD11b/c^+^RP-1^+^ cells) with no difference from APCP- and vehicle-treated group (**Figure 6A**). Moreover, the percentage of CD73^+^ cells among them (CD11b/c^+^RP-1^+^ CD73^+^) was very low in both groups (**Figure 6A**). Flow cytometric analysis of cells collected at 72 h showed that the main population within leucocytes were represented by monocytes/macrophages (CD11b/c^+^CD68^+^ cells) that were significantly reduced in pleural fluids harvested from APCP-treated rats compared with those from vehicle-treated rats (**Figure 6B**). In both vehicle- and APCP-treated groups, these cells were positive to CD73 (CD11b/c^+^CD68^+^CD73^+^cells) (**Figure 6B**). It is worth noting that another subset of cells, CD11b/c^+^CD68^-^, represented about 40% of cells harvested at 72 h.

## Discussion

There is much evidence for the role of CD73 in maintenance the integrity of epithelial barrier (Narravula et al., 2000; Salmi and Jalkanen, 2005; Bowser and Broaddus, 2016). Early papers investigating the involvement of CD73 in physiopathological conditions found that this enzyme was an innate protection in lung injury (Eckle et al., 2007). Pleural mesothelium is a monolayer of mesothelial cells lining the pleural cavity, presenting intermediate features between epithelium and endothelium; mesothelial cells participate to the pleural space homeostasis and represent the primary cells that initiate the response to an injury (Mutsaers, 2004; Batra and Antony, 2015).

Injection of carrageenan into the rat pleural cavity elicits an inflammatory response characterized by pleural effusion that dominates the early phase (4 h), characterized by cell recruitment, mostly PMNs, cytokine production, and tissue morphological changes. The model may resemble human pleural effusion, a severe complication often associated to pulmonary and extra pulmonary pathologies (Vinegar et al., 1982; De Brito, 1989; Murai et al., 2003; Lansley et al., 2017). Here, we have evaluated the effect of APCP, a CD73 inhibitor, in the inflammatory response triggered by carrageenan, in the model of rat pleurisy.

Following rat treatment with APCP, we found increased pleural effusion and total cell accumulation into the pleural cavity, in response to carrageenan injection, 4 h thereafter. At the same time point, the increased cell infiltration was paralleled by increased cytokine levels (IL-6, TNF alpha, IL-1 beta, MCP-1) into the pleural cavity. Conversely, in the pleural lavage fluids collected 72 h following injection of carrageenan, cytokine levels were almost absent in agreement with evidence of their early involvement in this model of inflammation (Goodman et al., 1993; Murai et al., 2003). Furthermore, at 72 h, there was no difference in cellular and cytokine content between vehicle- and APCP-treated group, apart from MCP-1 whose levels in APCP group were still higher than those in vehicle group.

It is known that MCP-1 represents a chemokine that, in the pleural space, is produced by mesothelial and inflammatory cells (Deshmane et al., 2009; Batra and Antony, 2015). Recently, Lansley et al. (2017) have demonstrated that MCP-1 plays a crucial role in pleural effusion in a mouse model of carrageenan-induced pleurisy. Here, we show that MCP-1 is the cytokine that prevails in pleural lavage fluids at 4 and 72 h following carrageenan injection in rats; moreover, our results suggest that MCP-1 production is under the control of CD73. The role of CD73 in the control of neutrophil influx in response to inflammation and infections has also demonstrated in experiments performed in transgenic mice lacking CD73 enzyme, in which following *Mycobacterium tubercolosis* infection, an increased influx of PMNs but not of monocytes has been observed (Petit-Jentreau et al., 2015). Consistently, we found that treatment with APCP at the onset of inflammation affects the early phase (4 h), dominated by neutrophil influx, but not the sub-acute phase (72 h) of inflammation, dominated by monocyte influx. APCP is a well described CD73 inhibitor; however, there is evidence that this compound also inhibits human nucleotide pyrophosphatase/phosphodiesterases (NPPs), a related nucleotidase hydrolizing ATP or ADP to AMP although with very low potency (Bhattarai et al., 2015). NPP is also involved in the adenosine pathway recognized as non-canonical, leading to adenosine accumulation from ATP and NAD (Ferrero et al., 2019). Furthermore, there is evidence that APCP through increasing AMP levels may indirectly inhibit NTPDase (CD39) (Covarrubias et al., 2016). Nonetheless, what we have found is that treatment with APCP exacerbates carrageenan-induced pleurisy in rats; we claim that to this effect contributes the inhibition of CD73, and probably of the other enzymes involved in the adenosinergic pathway, with the resulting loss of the control that these ectoenzymes exert on the inflammatory environment. Thus, CD73, or more extensively the adenosinergic pathway, results to be critical for PMN trafficking in the model of carrageenan-induced pleurisy in rat. Similarly, it has been demonstrated that CD73 controls PMN trafficking in lung injury induced by bacterial lipopolysaccharide inhalation in mice (Reutershan et al., 2009).

We also attempted to analyze cells collected at 4 h, to evaluate how they behaved far from the inflammatory microenvironment. For this purpose, we evaluated cell migration *ex vivo*, in presence or not of fMLP as chemoattractant agent. Interestingly, we found that PMNs collected from APCP-treated animals showed increased ability to migrate, even spontaneously, in absence of fMLP, compared to cells obtained from vehicle group. These results suggest that early inhibition of CD73 makes inflammatory cells, mostly neutrophils, prone to migrating. Likely, this effect may depend upon a contact with elevated levels of cytokines of cells obtained from APCP-treated animals compared to cells obtained from inflamed vehicle-treated animals. Alternatively, we might hypothesize that when CD73 is inhibited, a phenotypically different neutrophil population accumulates at the site of inflammation.

Neutrophil heterogeneity has been described (Scapini et al., 2016), and it is known that their differentiation toward distinct subpopulations depends upon environmental signals and also their lifespan (Silvestre-Roig et al., 2016). It is known that CD73 expression on neutrophils is normally low, and it’s under the control of inflammatory cytokines (Szabo and Pacher, 2012; Antonioli et al., 2013). By performing flow cytometric analysis of cells accumulated into the pleural space, we found that only a very small percentage of all leukocytes accumulated 4 h following carrageenan injections were CD73^+^, conversely, almost the totality of leukocytes accumulated at 72 h were CD73^+^, and there was no difference in the percentage of cells CD73^+^ between vehicle- and APCP-treated group. Nonetheless, there was difference in AMPase activity between cells obtained from the two groups, being the activity of cells recovered from pleural lavage fluid of APCP-treated rats significantly lower than AMPase activity of cells from vehicle-treated group. All together, these results suggest that cell trafficking into the pleural space is regulated by CD73 enzymatic activity. Our data agree with previous experimental results, in mice, and clinical data that highlight the important regulatory role of CD73 in the control of cell trafficking and cytokine production in an inflammatory environment (Reutershan et al., 2009; Petrovic-Djergovic et al., 2012; Al-Taei et al., 2016).

However, to better understand the mechanism by which CD73/adenosine pathway controls the inflammatory reaction in our model, we need to establish which cells are targeted by APCP. Indeed, we found that the effect of rat treatment with APCP was evident at 4 hours, when only a very small subset of infiltrating cells were CD73^+^; thus, it is conceivable that APCP does not exert its effect on recruited cells but on stromal cells and/or on other type of cells, such as dendritic cells, representing a first line of host defense and demonstrating to infiltrate the pleural space (Condon et al., 2011). It is known that dendritic cells expressing both CD39 and CD73 transform a proinflammatory ATP-rich microenvironment into an antinflammatory adenosine rich environment (Silva-Vilches et al., 2018). Unfortunately, we did not sort dendritic cells by flow cytometry; nonetheless, it can be argued that subset of cells CD11b/c^+^CD68^-^ found at 72 hours are dendritic cells; however, this point needs to be further investigated.

In conclusion, rat treatment with APCP exacerbates the early phase of inflammation by controlling pleural effusion and polymorphonuclear migration *in vivo* and *ex vivo*. Although we cannot rule out the possibility that APCP inhibits other ectoenzymes involved in the adenosinergic pathway, we argue that CD73 inhibition might play a major role. On this basis, we suggest that CD73 might represent a valid biomarker for pleural effusion besides being a potential target for novel therapeutic interventions.

## Data Availability

The datasets generated for this study are available on request to the corresponding author.

## Ethics Statement

All procedures were performed according to the Italian and European regulations (DL 26/2014) on the protection of animals used for experimental and other scientific purposes and were approved by Italian Ministry of Health.

## Author Contributions

EC, SM, and CC contributed to the study design, study conduct, and data analysis. EC and CC contributed to the data collection, data interpretation and drafting of the manuscript. EC, RC, AI, and CC revised the manuscript.

## Funding

This work was supported by the grant of University of Naples Federico II (Research Program 2017–2019; DR 409 del 7 Febbraio 2017).

## Conflict of Interest Statement

The authors declare that the research was conducted in the absence of any commercial or financial relationships that could be construed as a potential conflict of interest.
